# Negative effects of lifespan extending intervention on resilience in mice

**DOI:** 10.1371/journal.pone.0312440

**Published:** 2024-11-21

**Authors:** Katelynn M. Corder, Jessica M. Hoffman, Anamarija Sogorovic, Youfeng Yang, Anisha Banerjee, Yi Sun, Michael B. Stout, Steven N. Austad

**Affiliations:** 1 Department of Biology, University of Alabama at Birmingham, Birmingham, AL, United States of America; 2 Department of Biological and Environmental Sciences, Samford University, Homewood, AL, United States of America; 3 Department of Biological Sciences, Augusta University, Augusta, GA, United States of America; 4 Department of Medicine, Division of Gerontology, Geriatrics and Palliative Care, University of Alabama at Birmingham, Birmingham, AL, United States of America; 5 Department of Life, Health, and Physical Sciences, Gordon College, Wenham, MA, United States of America; 6 Aging and Metabolism Research Program, Oklahoma Medical Research Foundation, Oklahoma City, OK, United States of America; 7 Oklahoma City Veterans Affairs Medical Center, Oklahoma City, OK, United States of America; Arizona State University, UNITED STATES OF AMERICA

## Abstract

One key goal of basic aging research is the development of reliable assays of both current and future health. These assays could dramatically accelerate progress toward developing health-extending interventions by obviating the need for full lifespan studies, especially if they were informative relatively early in life. One potential approach is the assessment of physiological resilience, defined as the ability to recover from an adverse event. Here, using CB6F1 mice, we evaluated four potential resilience assays, each quantifying recovery from a physiological challenge with clear relevance to humans. The challenges were: (1) anesthesia recovery, (2) restoration of hemoglobin levels after a blood draw, (3) speed of wound healing, and (4) survival after pathogen exposure. We evaluated how each changed with age and with interventions known to extend health in males only (17α-estradiol) or both sexes (calorie restriction). We found that three of the four (recovery from anesthesia, blood draw, and pathogen exposure) showed significant and expected age effects, but wound healing did not. None of the three age-sensitive assays responded to the health-extending interventions in the way we expected, and for some assays, including anesthesia response, interventions actually worsened outcomes. Possible explanations are: (1) our interventions were too brief, (2) the ages we evaluated were too young, (3) our assays did not capture important features of organismal resilience, or (4) organismal resilience is not as clearly related to current or future health as hypothesized. Future studies are needed to determine which of these interpretations is valid and to determine whether other resilience metrics may be more informative about current and future health.

## Introduction

In response to the unprecedented aging of the global population and the attendant increase in chronic disabling diseases, healthcare costs, and concerns about the general quality of human life, research into the basic biology of aging, and the search for interventions that target that biology, have become ever more urgent. A major bottleneck in basic aging studies using laboratory mice, the workhorse of mammalian biomedical research, is the 3–4 years that it takes to complete longevity studies. Increased longevity is the gold standard for successful intervention in the biology of aging. Laboratory mice in well-managed colonies live as long as 27–30 months on average with the longest-lived animals surviving three years or more [1, 2]. Consequently, the development of inexpensive, simple, quick, and accurate early- or mid-life indicators of current and future health would not only alleviate this research bottleneck, but if these indicators were sufficiently general to also apply to humans, they would also dramatically accelerate progress in the search for, and clinical evaluation of, health-extending interventions that could have a major impact on global health.

Resilience, defined as the ability to recover from an adverse event or physiological challenge, is well-known to decline with age in humans and experimental animals [3–5]. This has been vividly shown recently by the dramatic age-related increase in COVID-19 hospitalizations, disability, and death [6], but COVID-19 is not exceptional in this regard. A similar pattern of significantly higher death rates among the elderly is seen in a broad spectrum of infectious diseases [7, 8]. Older people are also more likely to be hospitalized or die in heat waves, cold snaps, and due to traumatic injuries [9–11]. Therefore, quantitative measures of resilience seem a reasonable approach for the development of potential assays of current and future health in animals and humans [5, 12].

In this study, we sought to develop multiple simple, quick, easily standardizable, and inexpensive quantitative assays of resilience in mice. Each of the four assays we evaluated was based on common medical experiences of humans, so as to be as translatable as possible. The hope was that one, or some combination of, our assays would reflect current, and be predictive of, later health. Specifically, the four assays we evaluated were (1) recovery time following anesthesia, (2) rate of hemoglobin recovery after a blood draw, (3) rate of wound healing, and (4) response to a pathogen.

The rationale for the anesthesia recovery assay is that anesthetic complications, particularly neurocognitive disorders, increase with age in humans, and that recovery is slower with age in some inhalation anesthesia agents [13, 14]. Even when no statistical differences in recovery from anesthesia as a function of age are observed, there are consistent published trends toward longer recovery, and lack of statistical significance is often a consequence of large variation at any age [15]. A prominent geriatrics text notes that “physiological status is more important than chronological age” in recovery from anesthesia [16] suggesting this metric may be particularly informative about current and future health. In addition, recent work suggests there may be an association of response to anesthesia in middle age and longevity in mice [17].

Anemia is common among older people, and the risk for anemia increases with age [18, 19]. Therefore, we designed an assay to induce anemia based on blood draw, similar to what individuals of all ages experience. A standard 500 ml whole blood donation is about 10% of a person’s total blood volume. For safety reasons, donors cannot donate blood again until their hemoglobin (Hb) concentration has returned to a standard baseline value, which conservatively takes about 8 weeks (https://www.redcrossblood.org/donate-blood/how-to-donate/eligibility-requirements.html). The production of new red blood cells is a complex interaction of nutrient absorption by the intestines, kidney function (erythropoietin production), as well as the responsiveness of hematopoietic stem cells and erythroid precursors in the bone marrow [20]. Therefore, the rate of erythropoiesis and return to Hb homeostasis after a 10% blood draw might make an informative metric of a complex physiological function that typically declines with age.

Wound healing is another complex physiological process that can be assayed in a simple standardized fashion. It makes for an attractive assay as it is known to slow with age, involves multiple physiological systems, as well as the production and interaction of multiple cell types. Moreover, it involves processes known to be important in the biology of aging–inflammation and cell proliferation–which are decreased or delayed with age [21]. Intriguingly, previous research on calorie restriction indicates that at least one health-preserving intervention can enhance wound healing in older individuals [22].

Responses to infectious agents also vary with age, and declining immune defenses in both humans and other animals suggests such responses might be informative about current and future health. For instance, people over 80 years old infected with COVID-19 are roughly two hundred times more likely to die than people in their twenties [6]. Nor is this pattern limited to COVID-19, as a similar pattern is found for deaths from influenza [7]. Prior to the emergence of COVID-19, the leading cause of infectious disease death for those over age 65 was pneumococcal pneumonia due to *Streptococcus pneumoniae* [23]. Mice also get pneumonia from *S*. *pneumoniae*, and due to a long history of using mouse models to study this disease, a variety of *S*. *pneumoniae* strains are available with known virulence to mice [24]. We took advantage of this knowledge to develop a mouse pathogen challenge.

To investigate the utility of these resilience assays, we first assessed whether they declined with age in both sexes of CB6F1 mice acquired from the National Institute of Aging’s aged rodent colony (https://www.nia.nih.gov/research/dab/aged-rodent-colonies-handbook). We used an F1 hybrid model rather than an inbred strain, because individual mice are genetically identical like inbred strains, but the heterozygosity of F1’s at every locus where the parental strains differ makes them more robust and less prone to physiological idiosyncrasies compared to inbred strains [25].

Once we evaluated the impact of age on our four assays, we performed two intervention studies designed to help assess, by the sex-specificity of the results, the value of our assays as predictors of future mouse health. One intervention was dietary supplementation with 17α-estradiol (EST), a weakly feminizing estrogen found in multiple tissues of both sexes [26], which has been found to extend life up to 19% in male, but not female, mice even when started as late as 20 months of age [27, 28]. It has also been shown to have a number of health benefits, again in male mice only [26, 29]. The other was a calorie-restriction (CR) experiment. CR has long been known to enhance and extend health and life in both sexes of most laboratory mouse genotypes [30], including the CB6F1 mouse genotype we use here [2].

Given this design, our assays can be considered robust predictors of future health if singly or in combination, they reveal enhanced resilience in males only with EST supplementation but enhanced resilience in both sexes subjected to CR.

## Materials and methods

### Animals

All observations and experiments used CB6F1 mice, offspring of Balb/c females mated with C57BL/6 males. For most experiments we obtained known-age mice from the National Institute on Aging (NIA) aged rodent colony. However, for the CR experiments only, parental strains were purchased from Jackson Laboratory, and F1’s bred in our laboratory. CBF1 mice were chosen because they are a more robust animal model with one generation of outcrossing, but animals are still all genetically identical as they are derived from two highly inbred strains. All mice were allowed to habituate to our facilities for a minimum of two weeks before participating in experiments. Mating and rearing were done in the AAALAC-accredited animal facilities at University of Alabama at Birmingham.

All mice were housed in groups of 4–5 same sex animals unless otherwise noted. Mice were maintained at 21± 2°C ambient temperature with food and water provided *ad libitum* on a 12-hour light/dark cycle (lights on from 6:00 am to 6:00 pm). All experimental protocols were approved by the Institutional Animal Care and Use Committee (APN 10027) and were conducted in accordance with the Guide for the Care and Use of Laboratory Animals adopted by the National Institutes of Health. All observations and experiments were performed on mice between 12 and 25 months of age.

We should note that due to the limited availability of the mice from the NIA colony and the onset of the COVID-19 pandemic–and the subsequent restrictions that followed–the age groups used between experiments varied as well as which interventions were applied to each challenge. Within experiments, however, all mice were within two weeks of the stated age unless otherwise noted.

### Resilience assays

#### Anesthesia recovery

Mice were exposed to a continuous flow of 4% isoflurane for 10 minutes, which induced complete motionlessness. They were then removed from the induction chamber and placed dorsally on a warming mat. A camera recorded their recovery. We assessed several recovery metrics, settling on time-to-right–defined as time from placement in the recovery chamber to when the animal righted itself so that all four paws made contact with the floor–as our recovery metric, as it had the best interobserver consistency.

#### Hemoglobin recovery from blood draw

We drew ~10% blood by weight from each mouse (175–200 μl for a 25 g mouse) by retro-orbital bleed under light isoflurane anesthesia. Target blood draw volumes were calculated prior to the onset of experiments according to individual animal weight [31]. Subsequent, much smaller samples (~20 ul) were obtained, also via retro-orbital bleed, a maximum of 5 times over the following two weeks to assess recovery to baseline (from the original bleed) hemoglobin concentration using a HemoCue Hb 201 (HemoCue; Breas, CA). Blood draws were switched between eyes for each blood draw to allow sufficient healing time, and the starting eye was randomized for each mouse.

#### Wound healing

Mice were lightly sedated with 2% vaporized isoflurane, then received a 2mm diameter dermal punch biopsy with a clinical grade biopsy punch to the central part of each ear (S1 Fig). Body weight and the size of the wound of each ear were measured weekly post ear punch for 3–6 weeks under light sedation. Wound size was calculated by measuring the diameter of the circular wound with manual calipers along three axes and the measurements were averaged.

#### Pathogen challenge

We challenged lightly sedated (2% vaporized isoflurane) mice by intratracheal administration of 1x10^6^ CFU *Streptococcus pneumonia* strain TGR4 (gift of Dr. Carlos Orihuela) in 60μl saline and observed their recovery, as measured by body weight and survival. Sham controls were inoculated with saline using the same methods. Body weight and condition were recorded at baseline, directly after inoculation, and daily post-challenge for 12 days. Recovery time was the preferred measure, as indicated by a return to initial body weight and body condition score. However, many of the subjects became moribund after infection and this was used to indicate resilience to the pathogen. Humane endpoints were used when possible to alleviate suffering. Mice were checked 2–3 times per day and body weight and a Body Condition Score was noted. Mice were humanely euthanized using standard processes if certain indicators of distress are present including ruffled fur, unresponsive to stimulus, >20% weight loss, and signs of bacterial meningitis within two hours of being observed to have met the criteria. A total of 77 animals were used for this experiment (12 months: 13 females, 13 males; 18 months: 12 females, 13 males; 22 months:13 females, 13males). A total of 52 mice died or were euthanized after reaching the indicated threshold (12 months: 2 female, 9 male; 18 months: 6 female, 11 male; 22 months: 12 female, 12 male).

### Experimental interventions

We evaluated the response of our four resilience assays to two previously described life- and health-extending treatments, dietary 17α-estradiol (EST) supplementation and calorie restriction (CR).

#### 17α-estradiol dietary intervention

Twelve months old mice underwent baseline assessment of anesthesia and blood draw recovery just prior to randomization into control or intervention arms of the study. A control diet, TestDiet 58YP (66.6% carbohydrate, 20.4% protein, 13.0% fat) was supplemented with 17α-estradiol at 14.4 ppm to make the intervention diet. This is the same diet previously shown to extend male only life and health in UM Het3 mice [27, 32]. Diets were prepared by TestDiet, a division of Purina Mills (Richmond, IN). Both control and treatment groups were provided food and water ad libitum throughout the duration of the study. Resilience assays were repeated after 3 months and again after 8 months (mouse ages 15 and 20 months, respectively) after the introduction of the intervention diet.

#### *In vivo* body composition

*In vivo* body composition (total body fat) was measured using an EchoMRI™ 3-in-1 quantitative magnetic resonance (QMR) machine (Echo Medical Systems, Houston, TX). Following a system test using a known fat standard prior to the measurements being taken, mice were weighed and then placed into a clear holding tube capped with a stopper that restricted vertical movement, but allowed constant airflow. The tube was inserted into the machine and the measurement was performed.

### Behavioral assays

In addition to our resilience assays under EST supplementation, we also performed a battery of three simple, quick age-sensitive behavioral assays on the same animals. These were open field test, cage wire hang time, and nest building assessment. All of these assays are commonly used to measure different facets of rodent health. Prior to testing, animals were handled 4–5 minutes per day for at least two days to habituate them to the investigator. Animals were also habituated to the testing rooms for a minimum of 45 minutes before initiation of testing, and males and females were tested separately to mitigate the impact of the opposite sex being present during testing [33]. All behavioral tasks were performed as a battery over a 24-hour period for each animal. Apparatuses used were cleaned between each animal with 2% chlorhexidine and 70% ethanol following by a second cleaning with ethanol alone. Weights were recorded following testing.

#### Open field test

The open field test is often used to evaluate spontaneous locomotor activity and anxiety-like behavior, and locomoter activity declines with age while anxiety-like behavior increases with age in mice [34]. A camera was suspended above the testing apparatus–a box with opaque sides measuring (61 x 20 x 43 cm) in a lighted room. Mice were allowed to freely explore the open field for 5 minutes before being removed. Video recordings were analyzed using EthoVision software (Noldus, Leesburg, VA) to track all movements of each individual mouse. The field was divided into a 43 x 27 cm central region and a peripheral region. Total distance traveled in the entire field was measured to evaluate locomotor activity. Time spent in the central region was used to evaluate anxiety-like or exploratory behavior.

#### Cage wire hang task

The cage wire hang task was used to evaluate mouse four-limb strength and endurance. Muscle strength and endurance is well-known to decline with age in every species tested. The wire hang test has also been used to detect strength differences between young, middle-age, and old mice [35], as well as a measure of overall physical health. Mice were placed on a wire cage top suspended 38 cm above an open cage. The top was then gently shaken by the investigator to ensure the mouse gripped the wire before the top was inverted above the cage. A video camera recorded time until the mouse lost its grip, falling into its cage. If the mouse failed to remain on the cage top for more than 10 seconds, this trial was marked as a “fail”, and it was repeated. If the subject had more than 3 failed trials, data were removed for that trial. Two trials were conducted for each animal with > 10 minutes rest time between trials. Average latency to fall was analyzed.

#### Nest building assessment

Nest building behavior is important to mice for heat conservation as well as shelter and reproduction [36]. It is done by both sexes and is sensitive to differences in both age and sex [37, 38], and it is often considered similar to an activity of daily living in humans. Prior to the test, each cage was standardized for amount and depth of bedding (100g, approximately 0.5cm). Two hours prior to the onset of their dark phase (~4 pm), individually housed mice were provided with soft, unshredded cotton nesting material (4g, Ancare). Approximately, two hours after the onset of the following light cycle (~8 am), each cage was inspected for nest building progress and nests were photographed from multiple angles (S2 Fig). Unshredded nesting material was collected and weighed. Nests were scored based on previously published criteria [36] using a five-point system which takes into account the percentage of nesting material shredded and the overall shape and construction of the nest.

### Calorie restriction intervention

CB6F1 mice bred in our UAB colony were randomized into either *ad libitum* (AL) or 30% calorie restriction (CR) at 13–17 months of age. Animals were individually housed, and food intake was monitored daily for two weeks to establish baseline AL food consumption. The 30% CR diet was imposed in a stepwise manner over three weeks with 10% CR for two weeks and 20% CR for one week, and 30% restriction thereafter [39]. Eight weeks after implementation of 30% CR (10 weeks after beginning food reduction) resilience assays were started.

### Statistical analysis

All statistical analyses were completed using R 3.6.1 (www.r-project.org). For each resilience assay, we looked at the effects of age, sex, and the age-by-sex interaction controlling for body mass. We also ran a second set of analyses that were sex-specific. For anesthesia effects, we used a standard linear model. For the wound healing and blood draw assays, we ran a longitudinal model using R’s “nlme” package to determine the effects of our variables of interest, controlling for the effect of time (day of experiment), using Animal ID as a random effect. For the pathogen study, we ran a Cox Proportional hazard model on survival with pathogen administration. Animals were considered alive (0) or dead (1) after 12 days of experimentation. For the EST supplementation study and CR study, we analyzed the sexes separately. For the EST analyses, data were analyzed with a longitudinal model looking at the effects of age, diet, and the age*diet interaction, controlling for body weight with Animal ID as a random effect. For the blood draw assay in the EST supplementation experiment, we only analyzed the final (8 month, animals 20 months of age) timepoint, so as to avoid dealing with multiple longitudinal models of time since blood draw. One extreme outlier was removed from the estradiol anesthesia analysis. For CR analyses, we looked at the effects of diet controlling for body weight, as measures were not done longitudinally, and all animals were close in age. Missing data were removed from each individual analysis. Unless otherwise noted, p < 0.05 was considered significant for all tests. Plots were created with raw data in either GraphPad Prism or R.

## Results

### Age effects

We first assessed the impact of mouse age on each of our assays. We did this cross-sectionally, using three groups of known-age mice obtained from the NIA aging mouse rodent colony. Complete statistics for each test are in S1 Table.

**Fig 1:**
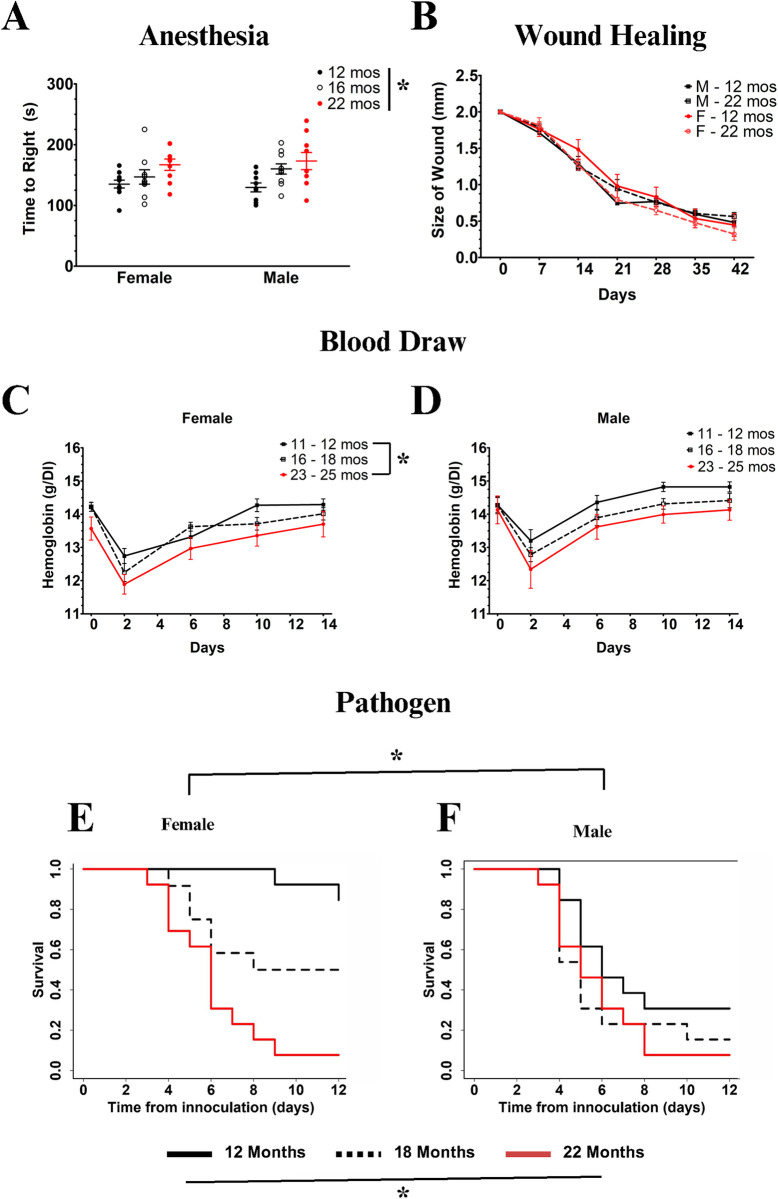
Impact of age on putative resilience assays. **(A)** Anesthesia recovery displays a significant increase with age (p = 0.004) but not sex (p = 0.36). **(B)** Ear punch wound healing does not change with age or sex. **(C, D)** Recovery from blood draw shows a trend towards an age effect (p = 0.081), but no impact of sex (p = 0.36). **(E, F)** Exposure to a standardized dose of *S*. *pneumoniae* pathogen reveals both age and sex effects on survival (p = 0.0009 for both). * = p <0.05.

For the anesthesia recovery assay, we assessed mice at 12, 16, and 22 months of age (Fig 1A). Controlling for body weight, since we did see an observable influence of body mass on recovery (S3 Fig) in males, we did observe an overall significant effect of age. Older animals took longer to right themselves after anesthesia (p = 0.004), but there was no significant effect of sex (p = 0.36). Analyzing each sex separately, there remained a significant age effect for both males (p = 0.016) and females (p = 0.022).

For healing of the 2 mm ear punch, we used animals of 12 and 22 months, finding no statistical difference in the trajectory of recovery (Fig 1B). On a separate group of animals in a pilot study, we also assessed recovery from a different wound previously reported in the literature [40], an interscapular biopsy punch, where we also found no vestige of an age effect.

For recovery from a blood draw, we assessed the entire trajectory from initial drop in Hb concentration to its recovery over a 14-day period (Fig 1C and 1D). Controlling for weight and amount of blood drawn, we found a trend towards an age effect (p = 0.081), with older animals showing lower hemoglobin concentration but no impact of sex (p = 0.26). Analyzing the sexes separately with the reduced statistical power that implies, we found that 22 month females significantly differed from 12 month females (p = 0.022) but no remaining age effect in males.

For resilience to the *S*. *pneumoniae* pathogen, we observed significant age and sex effects (p = 0.0009 for both) on survival after 12 days postexposure (Fig 1E and 1F). Specifically, male survival was worse than female survival, and older animals survived worse than younger animals. There was also a significant age-by-sex interaction (p = 0.005) in which females had a greater decline with age than seen in males.

### Effect of 17α-estradiol (EST) supplementation

As noted earlier, dietary supplementation with 14.4 ppm EST has been shown to extend life and health in male, but not female, mice [27, 29, 32]. Therefore, if our resilience assays were improved by EST supplementation in males only, it would be a strong indication that they were informative indicators of future health.

**Fig 2:**
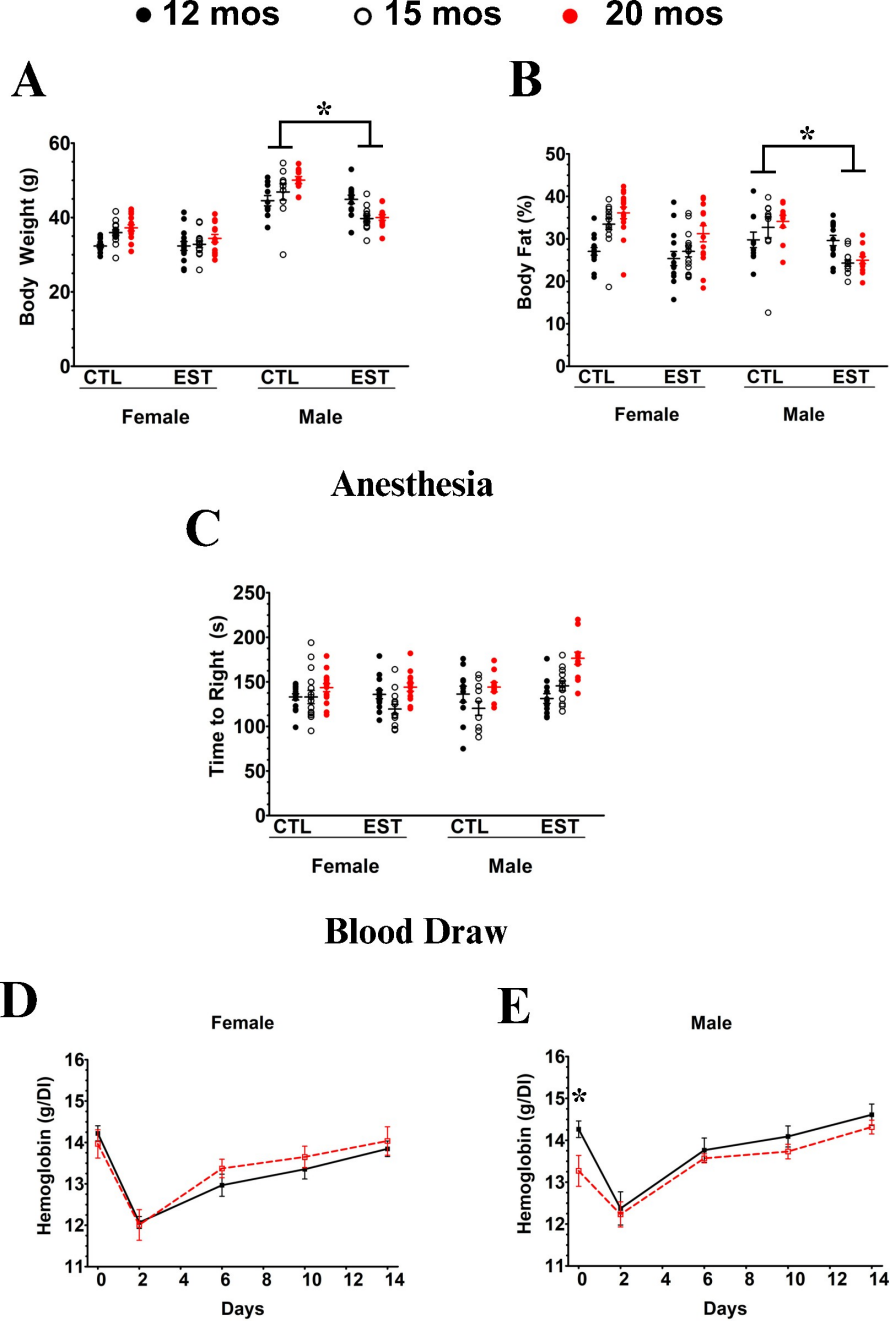
Impact of EST supplementation. Body weight **(A)** and percent body fat **(B)** at baseline (12 months old) and after 3 and 8 months of supplementation (15 and 20 months old, respectively). Note that EST-fed males never return to baseline body weight even though controls continue to gain weight. Effect seems to be due to reduced fat mass. There was no impact of EST feeding on female body weight or composition. Contrary to our hypothesis, EST-fed males **(C)** are not more resilient than females to anesthesia. For blood draw **(D & E)**, red dashed lines indicated EST-fed animals, and males showed significantly lower Hemoglobin levels after eight months EST treatment. * = p <0.05.

We initiated EST supplementation at 12 months of age and assayed animals after 3 and 8 months of supplementation (at ages 15 and 20 months). For these serial measures, the nature of the ear punch or pathogen challenge assays does not allow serial sampling, so only anesthesia recovery and blood draw assays were done. EST supplementation caused an initial loss of about 10% in body weight in both sexes; however, females’ weight had returned to baseline by three months of supplementation where it remained stable for the duration of the experiment. By contrast male weight never returned to baseline (p = 0.002) even though control males continued to gain weight throughout the experiment (Fig 2A). Quantitative magnetic resonance (EchoMRI™) assessment of body composition revealed that the persistent weight loss in males was due to loss of body fat (Fig 2B).

In our anesthesia recovery assay, we replicated our age effect (p = 0.019, Fig 2C). However, there was no sex effect of EST supplementation on recovery time from anesthesia (p = 0.47). After 8 months of treatment, males who weighed more had faster recovery from anesthesia (p = 0.03), but this result was independent of EST treatment (p = 0.81).

Hemoglobin recovery from a 10% blood draw showed a puzzling pattern. Females showed no significant impact of EST supplementation over the eight months of the study (Fig 2D). In males eight months of EST supplementation led to lower baseline hemoglobin levels (p = 0.0001), but there was no difference from controls in the rate of recovery (Fig 2E).

**Fig 3:**
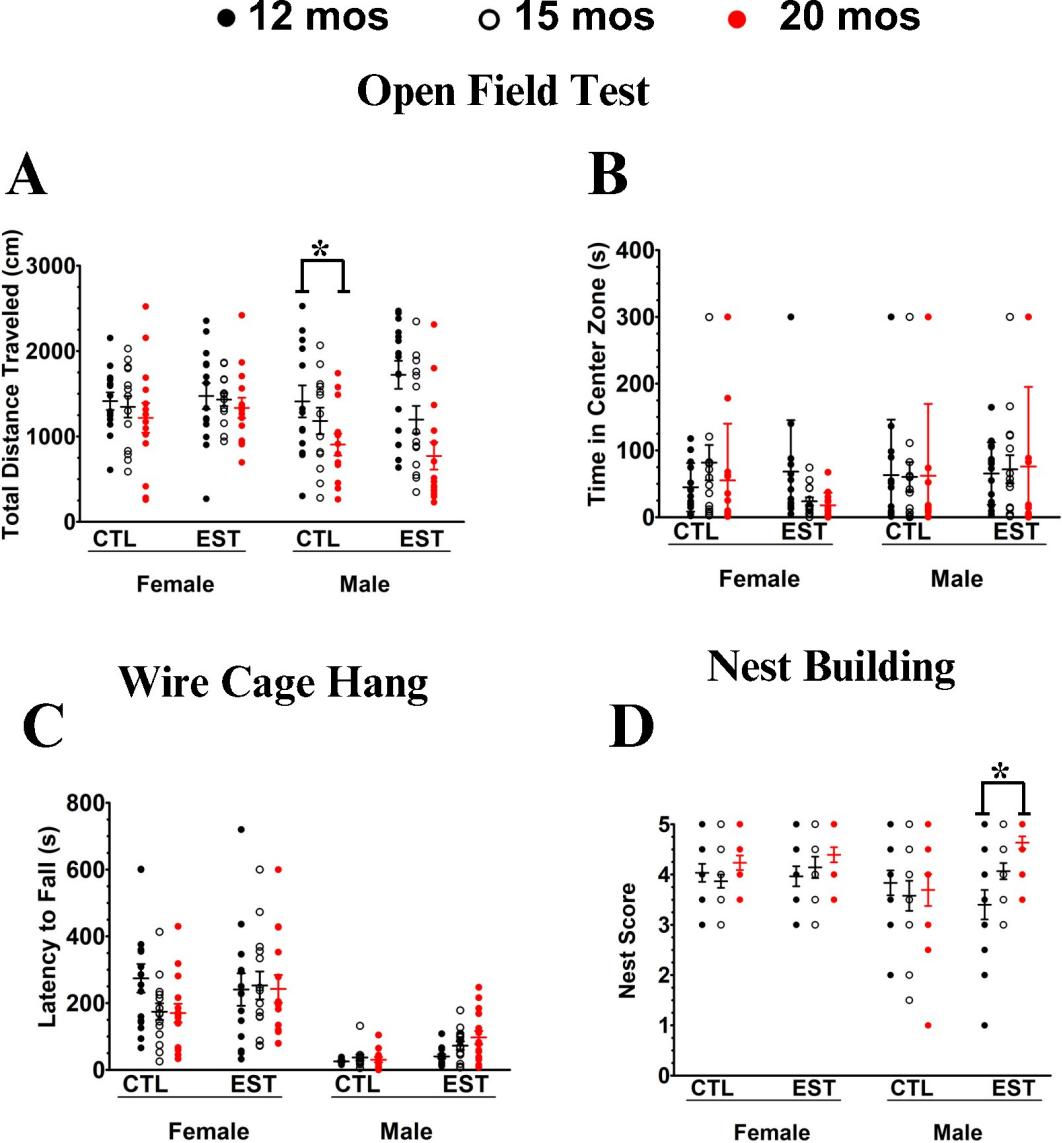
Behavioral impact of EST supplementation. **Open field (A, B).** There were provocative but very marginal sex-dependent effects of diet total distance traveled **(A)** (p = 0.09), but no trace of an effect of sex and diet interacting on time spent in the central zone **(B).** The cage wire hang test **(C)** did have a trend towards a (p = 0.07) sex-dependent effect of diet EST-fed males improving whereas females did not and a similarly marginal (p = 0.04) sex-dependent effect of diet on quality of nest building **(D)**. * = p <0.05.

Our behavioral assays associated with EST supplementation, the total distance moved in the open field was not affected by either age (p = 0.55) or diet (p = 0.99) in females, but in males, movement significantly decreased with age (p = 0.002) with a trend towards an effect of EST supplementation (p = 0.06). Males moved less than controls when EST was supplemented (Fig 3A). There were no significant effects of age or treatment on anxiety as determined by time spent in the center of the arena relative to the edges in either sex (p>0.18 for all). In the wire cage hang test, there was a trend towards (p = 0.07) sex-dependent effect with EST-fed males improving over time. Likewise, males showed a significant (p = 0.04) improvement in nest building when treated with EST, with no effects seen in females.

#### Effect of calorie restriction (CR)

**Fig 4:**
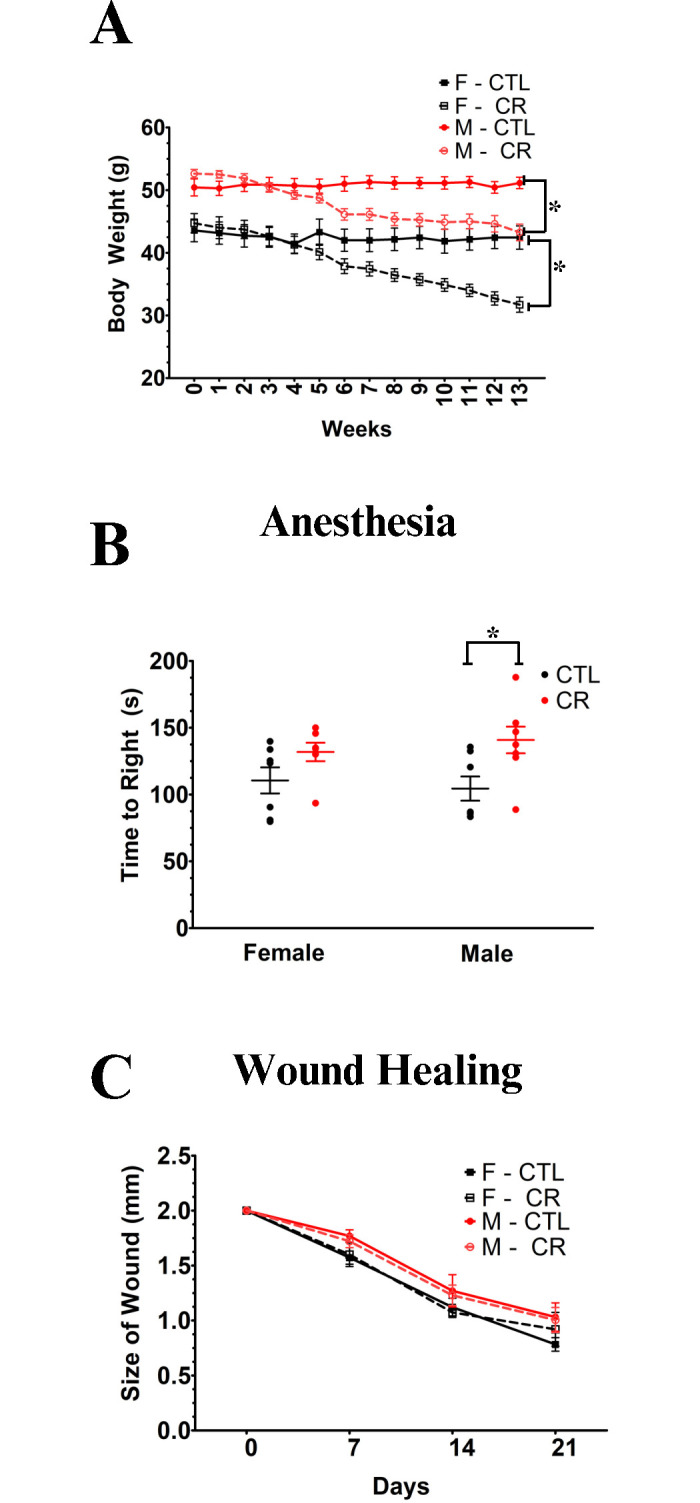
Impact of 8 weeks of calorie restriction (CR). **(A)** impact of CR on body weight. **(B)** Anesthesia recovery under CR was not statistically different from controls for either males or females (p = 0.14). **(C)** Wound healing under CR also did not differ significantly (p = 0.34) in either sex. * = p <0.05.

Calorie restriction is well-known to robustly extend life and health of both sexes in multiple genotypes of mice [2, 40]. As expected, our mice on 30% calorie restriction lost weight steadily over the course of the experiment (Fig 4A). We found a marginal effect of CR on recovery from anesthesia (p = 0.049) in males and a nonsignificant (p = 0.22) trend in the same direction in females (Fig 4B). However, both were in the direction of reduced, rather than enhanced, resilience–the opposite of what we expected. Similarly, we found no significant effects of CR on wound healing in either sex (p = 0.78 in males, p = 0.32 in females) and even the nonsignificant trends were in the direction of reduced rather than enhanced resilience.

## Discussion

To summarize, two of our four resilience assays (anesthesia recovery, pathogen challenge) showed robust age effects, one (recovery from blood draw) showed a statistically marginal trend in the direction of declining function with age, and the fourth (wound healing of an ear punch) showed no hint of an age impact. Our EST supplementation experiment did not show a general improvement in resilience in male mice only as our hypothesis predicted, if anything EST males performed worse on the resilience assays, but like previous studies, males were the only sex affected by the drug in several parameters. EST supplement did not affect male recovery from anesthesia or from blood draw, but it did lower baseline hemoglobin levels, suggesting that it may be having some negative physiological effects. It has been established previously, that 17β-estradiol can inhibit erythropoiesis [41], and our results suggest 17α-estradiol may also show similar phenotypes. In addition, EST had marginal impacts on male movement in the open field but in the opposite direction than expected if EST improved male health. Similar to previous reports [29], we found that EST-treated males, but not females, lost weight—largely body fat—during the experiment and never recovered to baseline, whereas control males continued to gain weight as they aged. There was no impact of EST on anesthesia recovery in females, but males at 8 months recovered more slowly, opposite to what our hypothesis predicted. EST also had no impact on recovery from a blood draw in females. However, males had a significant decrease in baseline hemoglobin levels on EST–opposite to our hypothesis. One behavioral test, quality of nest-building did show a positive effect of EST in males but not females, suggesting that this simple assay may be worth investigating further, as this is an indicator of overall mental and physical health of the animals [34, 42]. The other behavioral assays were not affected by EST supplementation in either sex. In our CR experiment, anesthesia recovery was worse, not better, in CR-treated mice of both sexes, again suggesting a negative physiological effect of the lifespan extending intervention.

All of our resilience assays except wound healing exhibited either a statistically significant, or trend, toward declining with age as expected. The age effect would no doubt have been greater if we had employed a wider age range, but we felt it to be unlikely that resilience assays done prior to 12 months of age would be informative about later life health and survival. Recent resilience work in mice found a similar age effect for anesthesia response [17].

The wound healing assay surprisingly did not show any hint of an age effect, even though age has been reported to slow wound healing in laboratory mice (and rats) previously [22, 43]. However, typically the animals in those experiments were considerably younger than our youngest animals, and wounds were full-thickness wounds in the interscapular area rather than ear punch wounds. For instance, Cohen and colleagues [43] found that full-thickness interscapular area wounds healed more quickly in young (6 mo) male C57BL/6 mice than in mature (15 mo) or old (26–27 mo) mice. We should note, however, that young rodents do not always show faster healing than older ones, in some species such as longer-lived *Peromyscus* mice, old mice heal faster [44] and in C57BL/6 and BALB/c mice young mice (1–2 months) have worse wound healing abilities than middle aged (12 month) mice [45]. We piloted the use of full-thickness interscapular wounds in our studies, finding that they worked well for C57BL/6 mice which could not reach the wounds with their paws to scratch at them, whereas our more supple CB6F1 mice could. CB6F1 mice were observed by investigators to regularly chew or scratch at wounds and were readily able to dismantle or escape splints. Thus, it should be noted that mouse genotype can affect *the feasibility* of doing certain assays. Ear punch wounds were particularly attractive alternative as a model of wound closing as they are often done routinely to individually identify animals in a cage. Ear punches do close partially, even in older animals, and the rate of ear punch closure in old mice has been associated with overall health in the animals [46]. However, to be most useful for measurement of age-related changes, and to clearly distinguish between wound contraction and wound closure, more sophisticated approaches such as the use of splints or scaffolds to stabilize wound area may be necessary [47], and we were using simple measures of size of the wounds without looking at histological changes. Potentially we would find similar rates of wound closure in middle and old age mice, but differences in the “new” tissues structure. Another disadvantage of wound healing protocols as possible markers of biological aging in individuals are that they are difficult to do multiple times longitudinally on the same animal.

We had great hopes for the use of our *S*. *pneumoniae* pathogen challenge assay. The same pathogen indeed shows the same adult survival pattern (declining with age, females surviving better at all ages) as in humans [48, 49]. Our original intention was to administer nonlethal doses and track weight loss and regain as a sign of illness and recovery. However, after piloting multiple *S*. *pneumoniae* strains and multiple doses, we were unable to identify a strain and dose combination that led to weight loss, but not death, in a substantial fraction of a study cohort. Like wound-healing, recovery from a pathogen challenge, even if successful as a predictor of later health and mortality, has the drawback that it cannot be done serially in individual animals so cannot be used longitudinally.

Our failure to find an impact of our interventions in the direction of increased resilience could be attributable to several things. It could have been that the interventions were not applied for a sufficient length of time, although at least in the case of EST supplementation, which was continued for eight months, that seems unlikely. Previous EST interventions for only 10–15 weeks showed beneficial effects on metabolic health as well as reduced inflammation in male C57BL/6 mice [29]. For CR, the hypothesis that the intervention was not sufficient in length is more plausible, in that the weight loss associated with CR had not yet stabilized after 13 weeks of restriction (Fig 4A). Still our putative resilience assays showed no signs of changes in the expected direction. It is also possible that the energy deficit of CR is inimical to wound healing and recovery from anesthesia. Some previous studies of wound healing in calorically-restricted animals have re-fed the animals prior to assessing healing and reported enhanced healing rates in re-fed CR animals compared with age-matched controls [22]. We did not do that. Still others however have failed to find that effect whether or not the restricted animals were re-fed [50, 51].

We were particularly surprised to observe no impact of CR on recovery from anesthesia. Pre-operative CR, even short 2–3 day CR, has been shown to robustly improve surgical outcomes in mice [52, 53]. Our results suggest that for these two interventions at least, healthspan and lifespan may not be correlated. An increasing number of studies are showing disconnects between lifespan, the number of days an individual lives, and healthspan, the period of “healthy” time an individual lives [54, 55], and our study may suggest similar results for at least some aspects of health with EST and CR interventions.

Finally, it is plausible that this particular set of resilience assays, even the ones that have a progressive decline with increasing age, are not useful for predicting future health outcomes. However, a recent complementary study of resilience in genetically heterogeneous mice did find that response an anesthesia challenge in middle age was associated with longevity, in females at least [17], again indicating that mouse genotype matters. On the other hand, our assays might be more predictive if started later in life or followed over a greater time interval. Certainly, mid-life predictors of later life health are worth continuing to pursue.

### Caveats

While our study points towards some interesting trends in age, sex, and their interaction as potential markers of resiliency in mice, our study is not without its limitations. First, we were not able to complete every assay in each intervention due to COVID-19 laboratory lockdowns as well as personnel changes during the pandemic; however, our results still suggest that overall lifespan extending interventions may not increase all aspects of health or resilience in mice. Second, while we use an F1 genotype, our results may not be applicable to all the natural genetic variation seen across species. Third, we only looked at middle aged to old mice with no young (3–4 month) group. Adding a young group may have allowed us to see more age effects; however, interventions with the most potential are usually started in middle age, leading us to choose this as the youngest group of animals. Fourth, our retro-orbital bleeds were done under very light anesthesia for comfort of the mouse. While at a dose much lower than in our anesthesia assay, this may have biased our results, and we may have been seeing age effects of the anesthesia as compared to the blood draw only. However, our rates of recovery were similar across groups with the largest changes seen in either baseline hemoglobin or the response to the large blood draw, suggesting our effects were most likely not caused by the anesthesia. Lastly, while our sample size was quite large for a mouse intervention study (~12–20 animals per group), we were still underpowered to detect all interaction effects, and future studies may need even large sample sizes to fully realize the effects of specific interventions on different markers of health.

### Conclusions

Here we have presented one of the first studies to look at recovery from a panel of challenges as potential predictors of health, primarily for allowing short-term evaluation of putative interventions in the future. A reliable set of relatively quick tests to screen for an intervention’s potential effect on healthspan would be invaluable to the aging research community. Interestingly some of the challenges we expected to show declines with age did not in our F1 hybrid background genotype. In addition, we found that our chosen interventions, EST supplementation and CR, failed to make significant improvements on many of these same challenges. These results suggest that individual facets of health may not be impacted by the same physiological processes equally. Thus, interventions that increase lifespan may not improve all aspects of health and resilience or that resilience as defined by this study may not be evaluating the health consequences like we previously predicted. The idea is that measures of resilience integrate several physiological factors to provide a more general measure of overall health. Future studies are needed to really tease apart the individual contributors to resilience, as well as potentially develop different challenges that better predict future health outcomes.

## Supporting information

S1 Table All statistical results from the three cohorts (age, EST, and CR). (XLSX)

S1 Fig Representative image of ear punch. (TIF)

S2 Fig Representative images of nests built during the nest building assessment. (TIF)

S3 Fig Effects of body mass on anesthesia recovery. Body mass was plotted against time to right during anesthesia recovery. A linear regression analysis revealed a significant effect of body mass on anesthesia recovery in males, with higher body mass resulting in quicker recovery times (Y = -3.415*X + 312.5, R^2^ = 0.1685, p = 0.0270). There was no significant effect in females (Y = 0.9558*X + 116.4, R^2^ = 0.03296, p = 0.3552).
(TIF)
