# Supplementary figures and images for: Negative effects of lifespan extending intervention on resilience in mice
Source: PLoS One. 2024 Nov 21;19(11):e0312440. doi: 10.1371/journal.pone.0312440 (PMC11581327; doi:10.1371/journal.pone.0312440)

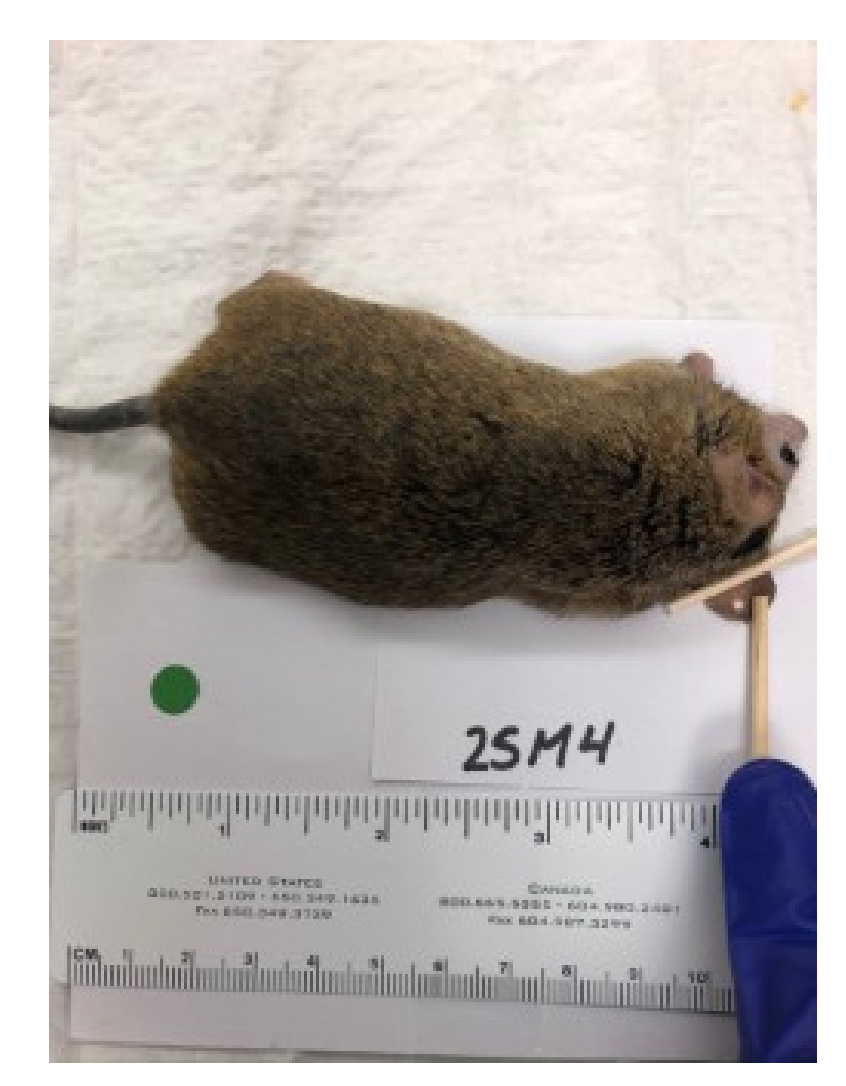

Supplement: S1 Fig — (TIF) [file pone.0312440.s002.tif]

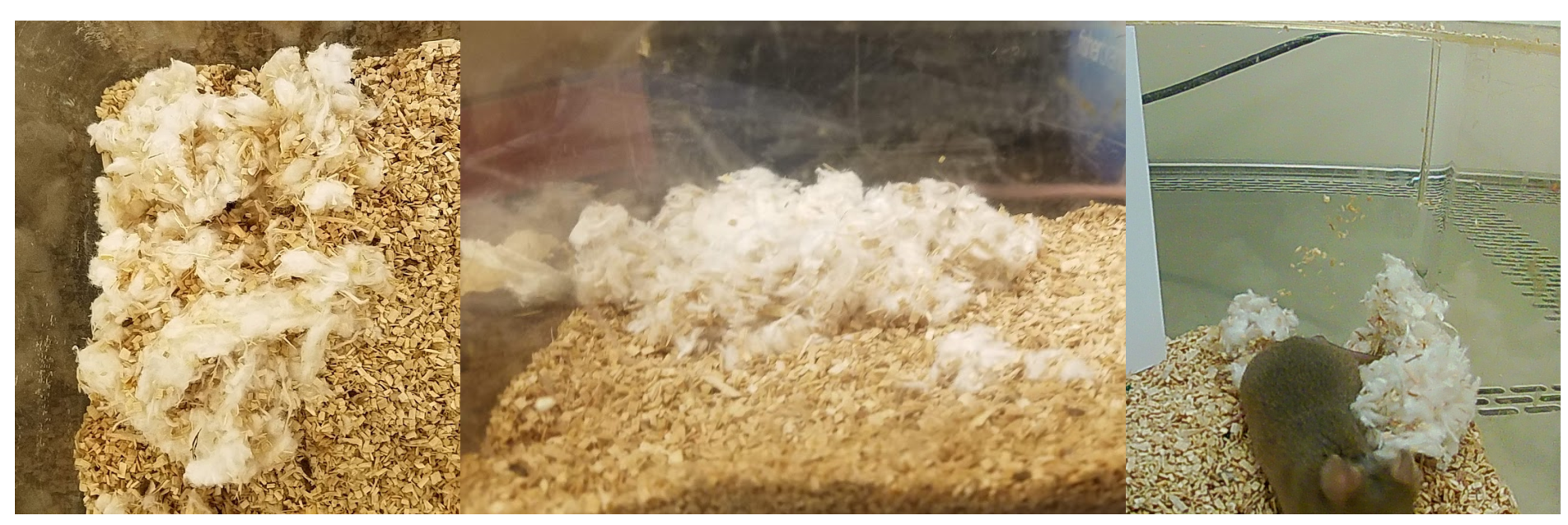

Supplement: S2 Fig — (TIF) [file pone.0312440.s003.tif]

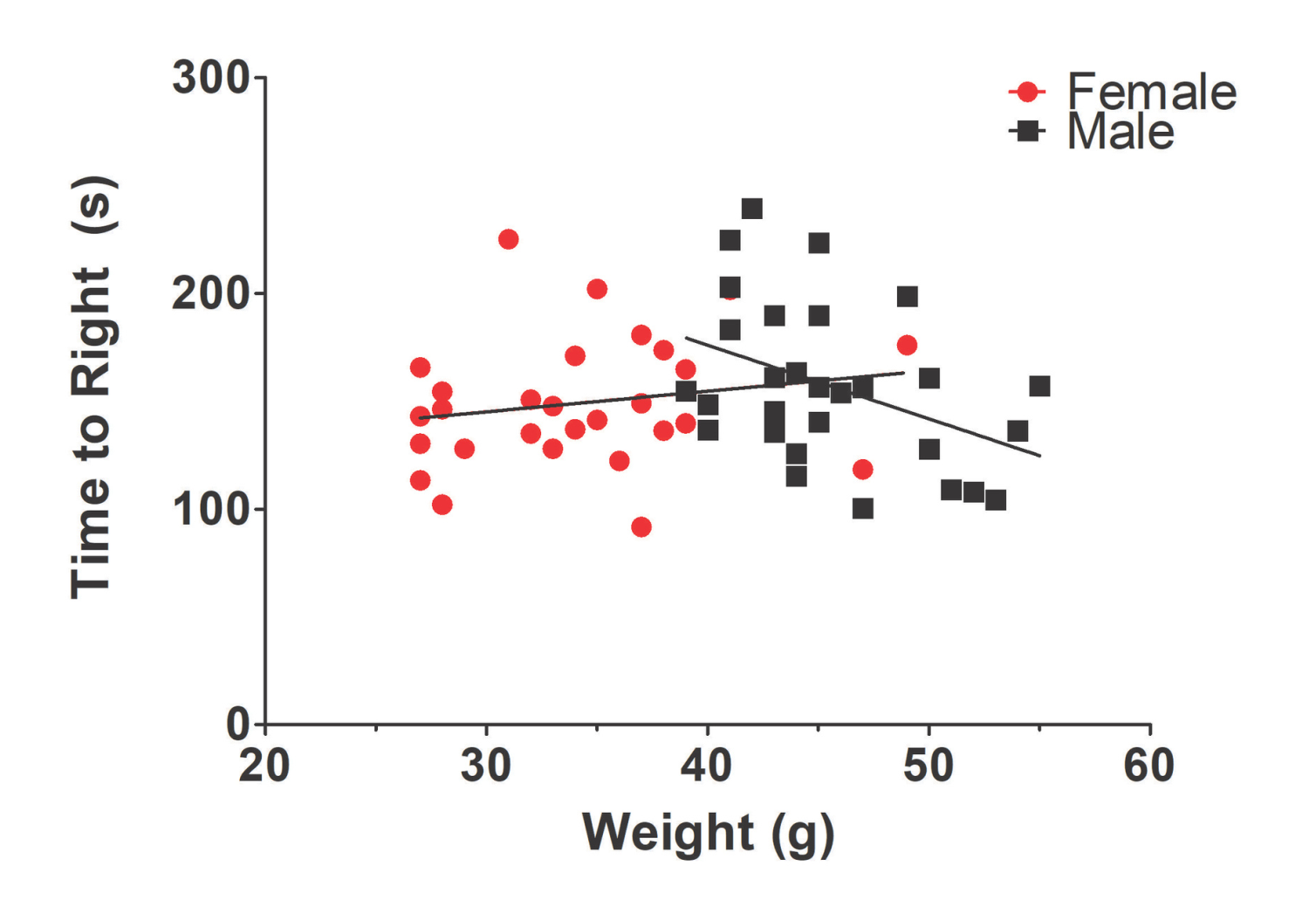

Supplement: S3 Fig — Body mass was plotted against time to right during anesthesia recovery. A linear regression analysis revealed a significant effect of body mass on anesthesia recovery in males, with higher body mass resulting in quicker recovery times (Y = -3.415*X + 312.5, R2 = 0.1685, p = 0.0270). There was no significant effect in females (Y = 0.9558*X + 116.4, R2 = 0.03296, p = 0.3552). (TIF) [file pone.0312440.s004.tif]
